# The impact of an unequal distribution of education on inequalities in life expectancy

**DOI:** 10.1016/j.ssmph.2021.100954

**Published:** 2021-11-04

**Authors:** Clemens Danler, Katharina Pfaff

**Affiliations:** a Department of Socioeconomics, Vienna University of Economics and Business, Welthandelsplatz 1, 1020, Wien, Austria; b Department of Socioeconomics, Vienna University of Economics and Business, D4.3.026, Welthandelsplatz 1, 1020, Wien, Austria

**Keywords:** Health disparity, Mortality, Knowledge acquisition, Life expectancy, Educational status

## Abstract

Prior research has found socio-economic determinants such as education to affect health outcomes. Yet, education is not distributed equally among the population. This article attempts to quantify the impact of unequal distribution of education on inequalities in life expectancy. We calculate a Gini coefficient of longevity from the life tables provided by the Human Mortality Database and a Gini coefficient of education using data on educational attainment from Barro and Lee (2013). We estimate linear regression models to examine the relationship between inequality in education and inequality in life expectancy at the country level for up to 31 European countries between 1970 and 2010. Results provide empirical evidence for a statistically significant positive association between educational inequality and inequalities of longevity. Confounding factors reflecting individual health behaviour such as cigarette or alcohol consumption do not exert a separate statistically significant effect on inequality in life expectancy. Findings are robust to alternative calculation of key variables, dropping a potential outlier, and an alternative estimation approach. These findings suggest that not only education, but also equality in education is a crucial factor for health outcomes. Continuing efforts should be directed towards the reduction of educational inequality in order to reduce inequality in longevity within a country.

## Highlights

• Educational differences between individuals predict differences in health outcomes.
• Cross-sectional research on the role of inequality is missing in this context.
• This study analyses whether educational inequality is linked to longevity inequality.
• Decreasing educational inequality decreases inequality in longevity.
• Results are robust for the sample of European countries.

## Funding statements

This research received no specific funding or other financial support. The authors are grateful for financial support of open access publishing by Vienna University of Economics and Business.

## 1 Introduction

Life expectancy has risen continuously in most high-income countries over the course of the 20th and 21st century (Ho & Hendi, 2018; Open & Vaupel, 2002). One crucial factor for explaining trends in longevity is education (Inequalities in Lon, 2017/02; Mackenbach et al., 2019; Cutler & Lleras-Muney, 2006). Education is inversely related to most major causes of death (Alicandro et al., 2018; Bijwaard et al., 2019; Nordahl et al., 2014) and health care access (Lazar & Davenport, 2018). Improving education provides individuals with a tool that increases their life expectancy both directly and indirectly (Mirowsky & Ross, 2003; Rogers et al., 2000). As the benefits of education grow over a lifetime and persist into old age (Mirowsky & Ross, 2003), increasing the level of education is of high relevance for policy makers.

Although there is much research on the positive impact of education on life expectancy, the influence of inequalities in education on lifetime inequality has not been examined so far. Yet, longevity is not evenly distributed within a society and likely to reflect socio-economic inequalities within a population. Knowing about the distribution of longevity across educational groups can provide policy makers with a more encompassing view about distributional patterns and suitable policies. Inequality in longevity, for instance, is significantly associated with greater market income inequality since income inequality is typically accompanied by a higher prevalence of poverty, which in turn can increase longevity inequality (Hill, 2018; Neumayer & Plümper, 2016). In this situation, not only public health policies could reduce longevity inequality but also income redistribution (Neumayer & Plümper, 2016).

Along similar lines, inequalities in education are directly and indirectly linked to inequality in life expectation. For instance, individuals with lower levels of education are associated with a higher body mass index (Brunello et al., 2016; Molarius et al., 2000) and obesity (Roskam et al., 2010). More education, in contrast, is associated with improved diets and more regular physical exercise (Brunello et al., 2016). In addition, lower educated individuals are not only more likely to develop alcohol abuse (Crum et al., 1993) and smoke (Escobedo & Peddicord, 1996; Gagné et al., 2015), but also less likely to attempt quitting to smoke as opposed to better educated individuals (Zhuang et al., 2015). Improved education is thus associated with a direct positive health impact.

Education also influences health and life expectancy through indirect channels. For one, education and related occupation class are a risk factor for non-fatal work injuries (Cutler & Lleras-Muney, 2006; Oh & Shin, 2003; Piha et al., 2013). While the causal relationship is challenging to disentangle, individuals with higher education may also be more likely to be employed in occupational classes, which pay higher wages and provide access to health insurance (Kawachi et al., 2010). These indirect channels matter since, for instance, uninsurance prevents individuals from meeting their health needs (Ayanian et al., 1993). Higher (minimum) wages, on the other hand, are related to a reduced likelihood of unmet medical needs (McCarrier et al., 2011). In addition to simply providing the financial means to address biological needs, higher income may increase longevity through societal effects as it allows individuals with to control their life circumstances (Chetty et al., 2016; van Raalte et al., 2011).

If education is unequally distributed within a population, this could also hint towards a spatial segregation of individuals with higher and lower level of education. Socioeconomic factors such as the educational level, for instance, explain part of the geographic variation in life expectancy among counties in the US (Dwyer-Lindgren et al., 2017). As a result, communities with lower average levels of education and, on average, lower life expectancy, could be spatially separated from communities with higher levels of education and resulting higher life expectancy.

In this research article, we fill a research gap and investigate the link between educational inequality and longevity inequality. If different groups within a population differ in their level of education, this will translate into differences in their health behaviour and, consequently, inequalities in life expectancy should be greater. In sum, we expect that lower inequalities in the level of education are associated with lower inequalities in life expectancy.

## 2 Methods

### 2.1 Dependent variable

We use the Gini coefficient to capture inequality (Neumayer & Plümper, 2016). The Gini coefficient indicates to what extent the Lorenz curve, which is a cumulative distribution function, differs from the diagonal line of perfect equality. The Lorenz curve can take on values between 0 and 1. The Gini coefficient of 0 denotes perfect equality. Perfect equality with respect to longevity means that all people were to reach exactly the same age i.e., 10% of the population would live exactly 10% of all years lived and 20% of the population would live exactly 20% of all years lived and so on. If a person had 100% of the total years lived, the Gini coefficient would equal 1 and reflect utmost inequality.

We use data from the Human Mortality Database (Shkolnikov et al., 2021) to calculate Gini coefficients of longevity for the entire range of ages. More specifically, we draw on the life tables from birth to age 110. Since child and adolescent mortality is increased, researchers sometimes use the data from the age of 15. However, as there are no significant differences between using the Gini of inequality from birth and from age 15, the data from birth onwards is used (Neumayer & Plümper, 2016). Although mortality data is available annually, we calculate 5-year intervals between 1950 and 2010 in line with the availability of our main independent variable. While 5-year intervals lead to a low number of observations, empirical results are not expected to differ significantly from those obtained with annual data or 3-year intervals (Neumayer & Plümper, 2016).

As the regular Gini index has difficulties to differentiate between different kind of inequalities and, for instance, the Gini of income inequality tends to be centred around 33% (Ultsch & Lötsch, 2017), we also use a standardized UGini index to better reflect the underlying distribution. The UGini is calculated as follows:

(1) $$r=\frac{b-a}{a}$$

where *r* is the relative difference between the values *a* and *b* which result in the new UGini when using non-logarithm Gini data. In other words, the formula describes the relative difference of the respective value to the uniform distribution. The transformation has the effect of reducing the density around mean values. Compared to the standard calculation of the Gini index, the mean of the UGini is centred around 0 instead of 0.11. A description of the sample is presented in Table 1.

**Table 1:** Summary statistics.

Variable	N	Mean	SD	Min	Max
Gini of longevity (baseline)	103	0.110	0.013	0.089	0.164
Gini of longevity (UGini)	103	0	0.120	−0.194	0.490
Life expectancy	103	74.062	8.174	16.22	79.84
Gini of education (standard, Barro-Lee)	103	0.155	0.097	0.007	0.503
Gini of education (UGini, Barro-Lee)	103	0	0.625	−0.955	2.235
External cause mortality rate	103	72.07	36.273	32.6	248.74
GDP PPP (log)	103	9.428	0.653	7.807	10.517
Health care expenditures to GDP (log)	103	1.701	0.310	1.001	2.413
Alcohol consumption per capita (log)	103	2.315	0.397	0.405	3.081
Cigarette consumption per capita (log)	103	7.402	0.339	6.360	7.991
Lung cancer mortality rate	103	37.365	11.992	12.09	67.32

### 2.2 Independent variables

#### 2.2.1 Educational inequality

To measure the distribution of education we use the education Gini coefficient (Galea & Ahern, 2005). The Gini of educational inequality was calculated using data from Barro and Lee (Barro & Lee, 2013). Their dataset provides data on educational attainment for 146 different countries from 1950 to 2010 in 5-year intervals. We use data for non-school education, for total, and for completed primary, secondary, and tertiary education of the population aged 15 and older. The following formula was used to calculate the Gini coefficients (Thomas et al., 2001):

(2) $$Gini=\left(\frac{1}{\mu }\right)\overset{n\;}{\underset{i=2}{\sum }}\;\overset{i-1}{\underset{j=1}{\sum }}p_{i}\;\left|y_{i}-y_{j}\right|p_{j}$$

where *p*_*i*_ and *p*_*j*_ are the proportions of population with a given level of schooling, y_i_ and y_j_ are the years of schooling at different educational attainment levels, μ is the average years of schooling for a given population and *n* is the number of levels/categories in attainment data.

#### 2.2.2 Additional covariates

We include the gross domestic product (GDP) per capita based on purchasing power parity (PPP) (Organisation of Economic, 2021) and health expenditures as share of GDP (Organisation of Economic, 2021) as inequality of longevity is negatively associated with the economic development of a country. In order to account for non-linear effects, both variables are logged and their second-degree polynomial terms included (Neumayer & Plümper, 2016). In order to account for factors of health risk behaviour, we also control for the logarithm of pure alcohol consumption in litres per year and person aged 15 and older, the number of cigarettes consumed per person per year, and mortality from lung cancer per 100.000 residents (World Health Organization, 2021). Please note that data on cigarette consumption is only available from 1970 to 2000, i.e. controlling for this control variable limits the time period of analysis and number of observations accordingly. In addition, we control for age-standardized mortality rates from all external causes of deaths such as accidents (Organisation of Economic, 2021). Furthermore, life expectancy at birth was included as a control variable in order to account for trends over time (Shkolnikov et al., 2021).

### 2.3 Descriptive analysis

**Fig. 1:**
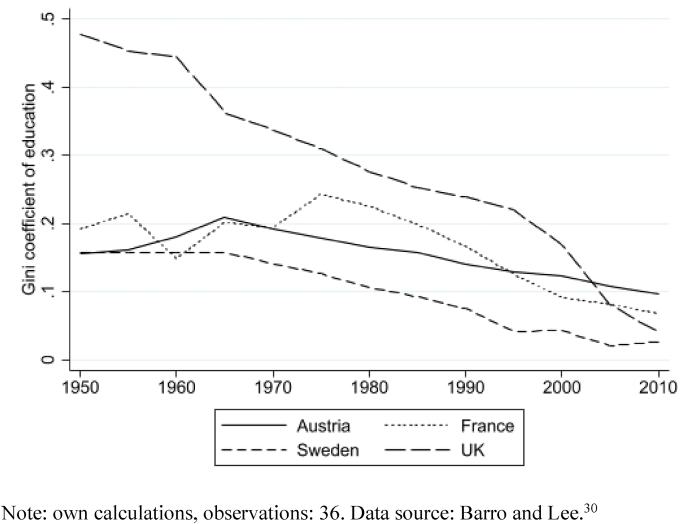
Gini coefficient of education from 1950 to 2010 in Austria, UK, France, and Sweden. Note: own calculations, observations: 36. Data source: Barro and Lee (Ultsch & Lötsch, 2017).

Fig. 1 displays the development of the educational Gini coefficient for selected countries over time. On average, the Gini coefficient decreased meaning that differences in the educational attainment of the population have become smaller. However, there are visible differences between countries, which have narrowed down over the years. As noted in Table 1, the mean education Gini in the sample studied was 0.11 (SD = 0.01, range = 0.08–0.16). The UK started with the highest educational inequality in 1970 but inequalities continually decreased until 2010. Sweden and Austria also experienced a decrease in inequality in education between 1970 and 2010. France had a short-lived increase in inequalities in education, but by 2010 the coefficients fall below the levels they had in 1970.

**Fig. 2:**
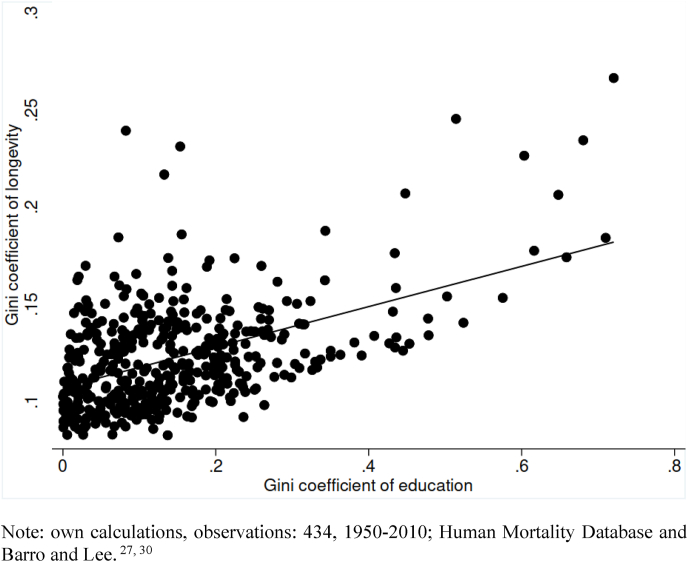
Correlation between inequalities in education and inequalities in longevity. Note: own calculations, observations: 434, 1950–2010; Human Mortality Database and Barro and Lee (Ultsch & Lötsch, 2017; van Raalte et al., 2011).

Fig. 2 depicts the correlation between the education inequality Gini index and the longevity Gini over the entire period 1950–2010 in all countries. Values of the inequality index of education are concentrated between 0.1 and 0.3 and of the Gini of longevity between 0.05 and 0.15. On average, higher inequality of longevity is observed in countries with more educational inequality at the different levels of education.

### 2.4 Data analysis

Due to data availability of the control variables, the analysis relies on a convenience sample of up to 31 countries with varying time frames from 1970 to 2010. The sample includes Israel as well as 30 European countries.^1^ We estimate the baseline sample with linear ordinary least squares regression. The Cumby-Huizinga test for autocorrelation shows that data are temporally dependent and thus likely to result in serially correlated errors. In order to counter this effect, a lagged dependent variable was included in the model. After the inclusion of the lagged dependent variable, the null hypothesis of no autocorrelation could not be rejected (Neumayer & Plümper, 2016). This supported by the coefficients of the lagged dependent variable as our estimation results show that they remain below the unit root threshold of 1. To account for country-specific heterogeneity, we also include country-fixed effects.

In addition to the linear regression analysis, we also estimate a linear dynamic panel-data model. This estimator is specifically designed for many panels (countries in our case) and few time periods (5-year intervals in our case). Furthermore, this model takes unobserved panel-level effects that are correlated with the lags of the dependent variable into consideration. This estimator requires no autocorrelation in the idiosyncratic errors. The Arellano–Bond test for no autocorrelation including the 5-year time periods source shows that the assumption for no autocorrelation is satisfied. The model was calculated using the xtabond command in STATA with the robust option enabled which makes the estimation robust to heteroskedasticity. In order to increase the sample size, the variable cigarette consumption was removed from these estimations.

## 3 Results

### 3.1 Main findings

Table 2 presents the estimation results with the Gini coefficient of longevity as the dependent variable, once for the ordinary least squares estimation and once for the linear dynamic panel data model. Results are very similar for both estimation approaches.

**Table 2:** Estimation results for Gini coefficient of longevity.

	Linear regression Coefficient (CI)	Linear dynamic model Coefficient (CI)
Lagged dependent variable	0.7624**	0.3637**
	(0.6460–0.8788)	(0.2050–0.5223)
Life expectancy	0.0000	−0.0000
	(-0.0000 - 0.0001)	(-0.0001 - 0.0000)
Educational inequality	0.0164*	0.0370**
	(0.0037–0.0291)	(0.0145–0.0595)
GDP PPP (log)	−0.0289**	−0.0036*
	(-0.0450 - 0.0129)	(-0.0065 - 0.0006)
GDP PPP (log) squared	0.0016**	
	(0.0007–0.0024)	
Health expenditures to GDP (log)	−0.0124	−0.0040
	(-0.0273 - 0.0024)	(-0.0097 - 0.0018)
Health expenditures to GDP (log) squared	0.0043	
	(-0.0002 - 0.0087)	
Alcohol consumption per capita (log)	0.0028	−0.0001
	(-0.0007 - 0.0063)	(-0.0068 - 0.0065)
Cigarette consumption per capita (log)	−0.0008	
	(-0.0031 - 0.0014)	
Lung cancer mortality rate (log)	0.0001	0.0000
	(-0.0000 - 0.0002)	(-0.0001 - 0.0002)
External cause mortality rate	0.0001**	0.0001**
	(0.0001–0.0002)	(0.0001–0.0001)

Number of countries	27	31
Time period	1970–2000	1970–2010
Country-fixed effects	Yes	No
Observations	103	141
R^2^/Wald χ^2^	0.99	1857.46

Notes: CI = confidence interval; GDP = gross domestic product. Constant included but not reported. The sample for the linear regression does not include Croatia, Estonia, Latvia, Luxembourg. ** statistically significant at 0.01 level, * at 0.05 level.

Educational inequality exerts, as expected, a positive and statistically significant effect on inequality in life expectancy. A one-unit increase in the Gini of education increases the Gini of longevity by 0.0164 or 0.0370, respectively. Life expectancy does not exert a statistically significant effect on inequality in longevity.

GDP has a negative statistically significant effect, i.e., the higher the gross domestic product, the lower the inequality in life expectancy. The nonlinear effect turns positive at higher levels of GDP. This can be interpreted that at low levels of the gross domestic product an increase of the gross domestic product is more effective in the reduction of the Gini of longevity. The higher the gross domestic product becomes, the smaller is the positive effect of an additional increase of one unit in the reduction of the Gini of longevity.

Health expenditure as a percentage of the gross domestic product also displays a negative marginal effect at lower levels of spending for healthcare expenses, which turns into positive values at a higher level of spending. These findings suggest that most countries in the sample already have high levels of health care spending, so that any further increase will primarily benefit the already elderly population and thus might increase longevity inequality (Neumayer & Plümper, 2016). Both coefficients are statistically not significant.

Alcohol consumption shows statistically insignificant effects on inequalities in longevity in both estimations. Our findings are supported by prior research (Grittner et al., 2013), which attributes higher, non-abusive levels of alcohol consumption to more highly educated persons and either complete abstinence from alcohol or unhealthy consumption to lower educated persons, which results in no clear correlation. The effect of cigarette consumption is close to zero and statistically insignificant. While the effect of lung cancer death rates is positive as expected, the coefficient is statistically insignificant. Finally, a one-unit increase in the external cause mortality rate provides a statistically significant increase in the Gini of longevity in both models.

### 3.2 Robustness checks

Table 3 provides further estimation results to assess whether our results are robust to an alternative calculation of the Gini index (UGini). Thus, all regression models presented in this study were also calculated with the UGini of longevity and UGini of education. The models are robust to the UGini calculations. Coefficients are similar and the level of significance remains unchanged or increased.

**Table 3:** Estimation results for Gini coefficient of longevity (UGini calculations).

	Linear regression Coefficient (CI)	Linear dynamic model Coefficient (CI)
Lagged dependent variable	0.7624**	0.4348**
	(0.6460–0.8788)	(0.2535–0.6162)
Life expectancy	0.0000	−0.0004
	(-0.0004 - 0.0005)	(-0.0010 - 0.0002)
Educational inequality (UGini)	0.0231*	0.0450**
	(0.0052–0.0411))	(0.0147–0.0752)
GDP PPP (log)	−0.2627**	−0.0171
	(-0.4086 - 0.1168)	(-0.0581 - 0.0239)
GDP PPP (log) squared	0.0141**	
	(0.0062–0.0220)	
Health expenditures to GDP (log)	−0.1130	−0.0232
	(-0.2479 - 0.0220)	(-0.0910 - 0.0447)
Health expenditures to GDP (log) squared	0.0386	
	(-0.0019 - 0.0791)	
Alcohol consumption per capita (log)	0.0252	0.0024
	(-0.0063 - 0.0567)	(-0.0696 - 0.0743)
Cigarette consumption per capita (log)	−0.0076	
	(-0.0280 - 0.0127)	
Lung cancer mortality rate (log)	0.0006	0.0011
	(-0.0002 - 0.0015)	(-0.0007 - 0.0030)
External cause mortality rate	0.0010**	0.0009**
	(0.0005–0.0015)	(0.0005–0.0012)

Number of countries	27	31
Time period	1970–2000	1970–2010
Country-fixed effects	Yes	No
Observations	103	141
R^2^/Wald χ^2^	0.99	2384.71

Notes: CI = confidence interval; GDP = gross domestic product. The sample for the linear regression does not include Croatia, Estonia, Latvia, Luxembourg. Constant included but not reported. ** statistically significant at 0.01 level, * at 0.05 level.

To examine the importance of considering educational inequalities, we compare our baseline model with a model, in which the Gini of education has been dropped. Once the Gini of education is removed from the model, the number of observations increases. Alcohol and cigarette consumption as well as health expenditure become statistically significant (see Table 4). At the same time, the explanatory power captured by the R^2^ slightly decreases from 0.99 and to 0.98, while the root mean squared error increases from 0.00141 to 0.00173. The statistically significant p-value of a Wald test, which assesses whether the coefficients for educational inequality, cigarette consumption and alcohol consumption are simultaneously equal to zero, suggests that these variables create an improvement in model fit.

**Table 4:** Estimation results without Gini coefficient of education (baseline).

	Linear regression Coefficient (CI)
Lagged dependent variable	0.6988**
	(0.6420–0.7555)
Life expectancy	−0.0000
	(-0.0000 - 0.0000)
GDP PPP (log)	−0.0084**
	(-0.0115 - 0.0053)
GDP PPP (log) squared	0.0003**
	(0.0002–0.0005)
Health expenditures to GDP (log)	−0.0098**
	(-0.0158 - 0.0039)
Health expenditures to GDP (log) squared	0.0022*
	(0.0004–0.0041)
Alcohol consumption per capita (log)	0.0028**
	(0.0010–0.0047)
Cigarette consumption per capita (log)	0.0015**
	(0.0004–0.0026)
Lung cancer mortality rate (log)	0.0001
	(-0.0000 - 0.0002)
External cause mortality rate	0.0000**
	(0.0000–0.0000)

Number of countries	27
Time period	1970–2004
Country-fixed effects	Yes
Observations	447
R^2^/Wald χ^2^	0.98

Notes: CI = confidence interval; GDP = gross domestic product. Constant included but not reported. ** statistically significant at 0.01 level, * at 0.05 level.

In public health literature, Portugal stands out for being a developed country with high health inequalities, which researchers have linked to educational inequalities (Campos-Matos et al., 2016). It is thus not surprising that in our sample Portugal is the country with the highest inequality in longevity and the highest educational inequality. As an additional robustness check, we therefore dropped Portugal as a potential outlier from our sample to examine whether results are exclusively driven by Portuguese observations. As reported in Table 5, educational inequality is still positive and statistically significant. The size of the coefficient is only marginally reduced and thus robust.

**Table 5:** Estimation results for Gini coefficient of longevity (excluding Portugal).

	Linear regression Coefficient (CI)	Linear dynamic model Coefficient (CI)
Lagged dependent variable	0.6398**	0.3375**
	(0.5334–0.7462)	(0.1502–0.5247)
Life expectancy	0.0000	−0.0000
	(-0.0000 - 0.0000)	(-0.0001 - 0.0000)
Educational inequality	0.0109*	0.0358**
	(0.0001–0.0216)	(0.0142–0.0573)
GDP PPP (log)	−0.0240**	−0.0036*
	(-0.0376 - 0.0103)	(-0.0070 - 0.0003)
GDP PPP (log) squared	0.0012**	
	(0.0005–0.0020)	
Health expenditures to GDP (log)	−0.0075	−0.0053*
	(-0.0218 - 0.0069)	(-0.0104 - 0.0002)
Health expenditures to GDP (log) squared	0.0022	
	(-0.0020 - 0.0065)	
Alcohol consumption per capita (log)	0.0004	−0.0017
	(-0.0026 - 0.0035)	(-0.0081 - 0.0048)
Cigarette consumption per capita (log)	−0.0003	
	(-0.0022 - 0.0016)	
Lung cancer mortality rate (log)	0.0000	0.0001
	(-0.0000 - 0.0001)	(-0.0001 - 0.0002)
External cause mortality rate	0.0001**	0.0001**
	(0.0001–0.0002)	(0.0001–0.0001)

Number of countries	26	30
Time period	1970–2000	1970–2010
Country-fixed effects	Yes	No
Observations	97	136
R^2^/Wald χ^2^	0.99	1359.58

Notes: CI = confidence interval; GDP = gross domestic product. Both samples exclude Portugal. The sample for the linear regression does also not include Croatia, Estonia, Latvia, Luxembourg. Constant included but not reported. ** statistically significant at 0.01 level, * at 0.05 level.

## 4 Discussion

In this paper, we tested the hypothesis that greater inequalities in education are linked to greater inequalities in longevity. Based on a cross-section of up to 31 countries from 1970 to 2010, we cannot reject our hypothesis. Controlling for several confounding factors related to life expectancy, we found empirical evidence that greater educational inequalities are positively and statistically significantly associated with greater inequalities of life expectancy. This evidence is robust to the use of an alternative estimator, an alternative calculation of the Gini index of inequality, and an outlier elimination test. Our study is the first study across countries that estimated the link between educational inequalities and inequality in the years lived at the country level. Findings directly highlight the importance of equality in education for health equality.

Our findings also provide tentative insights into the relative importance of education in reducing inequalities in life expectancy. Control variables reflecting individual health behaviour such as alcohol and cigarette consumption exert a marginal impact and are statistically insignificant. Educational inequality, in contrast, exerts an effect, which is separate from our proxy variables of health behaviour. This does not imply that a reduction in tobacco and alcohol consumption is ineffective in reducing mortality at the individual level. Our studies, however, provides no evidence that these variables are strong predictors of inequality in longevity once educational inequality is accounted for.

Prior research has provided robust evidence that policies reducing market income inequality reduce inequality in longevity (Neumayer & Plümper, 2016). While the effects of income may become apparent once an individual enters working age, education can exert positive effects earlier in an individual's life. An unequal education system not only disadvantages individuals with respect to their income or job opportunities. Education influences lifestyle choices and health behaviour from childhood onwards and thus influences the life expectancy at the individual level for a longer time span (Mirowsky & Ross, 2003). The present study provides evidence that inequalities in education can foster disparities within a country such that they are reflected in inequalities in life expectancy.

### 4.1 Limitations

The explanatory power of our results is limited by three main aspects. One limitation lies in limited data availability. First, our analysis cannot capture most recent developments as our data source only provides information on educational attainment by age groups until 2010 (Barro & Lee, 2013). Second, information on additional control variables is missing. Prior research has identified overweight or physical activity to be significantly related to the educational level and to be a high-risk factor for a shortened life expectancy (Brunello et al., 2016; Mirowsky & Ross, 2003; Molarius et al., 2000). While these are relevant factors, these variables could also not be included in our model due to issues of data availability at the country level. Third, data availability restricts our sample to a subgroup of European countries. Generalizations to non-European countries cannot be drawn.

Also, it has to be noted that this study uses observational data for a multivariate regression analysis at the country-year level. A causal mechanism through which educational inequality is affecting inequality in longevity was not directly tested and there is no evidence that the results can be applied to countries that are outside of the investigated sample. Strictly speaking, our findings merely highlight an ecologic association between the Gini coefficient of educational inequality and inequality in life expectancy. This positive association may reflect either a contextual effect of educational inequality on longevity, or a compositional effect of low-educated individuals residing in unequal states, or both.

## 5 Conclusion

Research in public health has provided ample proof for a positive relationship between education and life expectancy. Our cross-country study provided additional evidence that educational inequality has a statistically significant association with inequality in longevity. Countries, whose population have unequal access to education, are also more unequal in longevity. Against the backdrop of prior research highlighting the importance of education (Inequalities in Lon, 2017/02; Mackenbach et al., 2019; Cutler & Lleras-Muney, 2006), we believe that this is an important finding for policy makers. We recommend policy makers to address educational inequalities when intending to flat out inequalities in longevity.

Equal access to education, fair evaluation of performance, and an inclusive school system that does not favour people of certain socioeconomic background are factors that can be influenced by adequate policies. The standardisation of education, examination procedures, and grading systems can create a uniform standard and equal opportunities if the way in which performance is assessed are objective and do not carry forward existing inequalities. Especially when intergenerational educational mobility within a country is not perfect, educational policies are a powerful public health tool which individuals can profit from throughout their lifetime.

## Author statement

Clemens Danler: Conceptualization, Methodology, Investigation, Software, Formal analysis, Visualization, Writing - Original Draft.

Katharina Pfaff: Methodology, Writing - Original Draft, Writing - Review & Editing, Visualization, Supervision, Funding acquisition.

## Ethical statement

This study is based on cross-sectional observational data. There are no ethical aspects to declare.

## Declaration of competing interest

The authors have no conflicts of interest to declare.

## Footnotes

1 The full sample encompasses the following countries: Austria, Belgium, Bulgaria, Croatia, Czech Republic, Denmark, Estonia, Finland, France, Germany, Greece, Hungary, Iceland, Ireland, Israel, Italy, Latvia, Lithuania, Luxembourg, Netherlands, Norway, Poland, Portugal, Russia, Slovakia, Slovenia, Spain, Sweden, Switzerland, Ukraine, and United Kingdom.
